# Deep Seated Tumour Treatments With Electrons of High Energy Delivered at FLASH Rates: The Example of Prostate Cancer

**DOI:** 10.3389/fonc.2021.777852

**Published:** 2021-12-23

**Authors:** Alessio Sarti, Patrizia De Maria, Giuseppe Battistoni, Micol De Simoni, Cinzia Di Felice, Yunsheng Dong, Marta Fischetti, Gaia Franciosini, Michela Marafini, Francesco Marampon, Ilaria Mattei, Riccardo Mirabelli, Silvia Muraro, Massimiliano Pacilio, Luigi Palumbo, Loredana Rocca, Damiana Rubeca, Angelo Schiavi, Adalberto Sciubba, Vincenzo Tombolini, Marco Toppi, Giacomo Traini, Antonio Trigilio, Vincenzo Patera

**Affiliations:** ^1^ Dipartimento di Scienze di Base e Applicate per l’Ingegneria, Sapienza Università di Roma, Roma, Italy; ^2^ Istituto Nazionale di Fisica Nucleare (INFN) Sezione di Roma I, Roma, Italy; ^3^ Scuola post-laurea in Fisica Medica, Dipartimento di Scienze e Biotecnologie medico-chirurgiche, Sapienza Università di Roma, Roma, Italy; ^4^ Istituto Nazionale di Fisica Nucleare (INFN) Sezione di Milano, Milano, Italy; ^5^ Dipartimento di Fisica, Sapienza Università di Roma, Roma, Italy; ^6^ Unità di Fisica Sanitaria, Azienda Ospedaliero-Universitaria Policlinico Umberto I, Roma, Italy; ^7^ Museo Storico della Fisica e Centro Studi e Ricerche “E. Fermi”, Roma, Italy; ^8^ Dipartimento di Scienze Radiologiche, Oncologiche e Anatomo Patologiche, Sapienza Università di Roma, Roma, Italy; ^9^ Istituto Nazionale di Fisica Nucleare (INFN) Sezione dei Laboratori di Frascati, Roma, Italy

**Keywords:** external beam radio therapy, prostate cancer, FLASH effect, very high energy electrons, deep seated tumours

## Abstract

Different therapies are adopted for the treatment of deep seated tumours in combination or as an alternative to surgical removal or chemotherapy: radiotherapy with photons (RT), particle therapy (PT) with protons or even heavier ions like ^12^C, are now available in clinical centres. In addition to these irradiation modalities, the use of Very High Energy Electron (VHEE) beams (100–200 MeV) has been suggested in the past, but the diffusion of that technique was delayed due to the needed space and budget, with respect to standard photon devices. These disadvantages were not paired by an increased therapeutic efficacy, at least when comparing to proton or carbon ion beams. In this contribution we investigate how recent developments in electron beam therapy could reshape the treatments of deep seated tumours. In this respect we carefully explored the application of VHEE beams to the prostate cancer, a well-known and studied example of deep seated tumour currently treated with high efficacy both using RT and PT. The VHEE Treatment Planning System was obtained by means of an accurate Monte Carlo (MC) simulation of the electrons interactions with the patient body. A simple model of the FLASH effect (healthy tissues sparing at ultra-high dose rates), has been introduced and the results have been compared with conventional RT. The study demonstrates that VHEE beams, even in absence of a significant FLASH effect and with a reduced energy range (70–130 MeV) with respect to implementations already explored in literature, could be a good alternative to standard RT, even in the framework of technological developments that are nowadays affordable.

## 1 Introduction

The treatment of deep seated tumours with external beam radiotherapy (EBRT) is an effective therapy either in alternative or in addition to surgical removal or chemotherapy. While radiotherapy (RT) with photons is the reference treatment, the number of Particle Therapy (PT) centres delivering protons and carbon ion treatments is steadily increasing in time. Very high energy electrons (energies above a minimum of ~ 50 MeV are needed for an electron to reach a ‘deep’ seated tumour) have been considered in the past. In this paper we evaluate the potential of high energy electron beams according to the recent developments in the field of electrons acceleration and delivery, studying in detail the case of prostate cancer treatment. Prostate cancer has become the third most common cancer in men (nearly 10% of all cancers) and is the fifth leading cause of death worldwide (1, 2).

Early stages of prostate cancer can be treated using Intensity Modulated Radiation Therapy (IMRT) (3–6). One of the IMRT intrinsic limitations is related to how photons interact with the patient body. The absorbed dose peaks 1 or 2 cm (depending on the energy) after entering the patient body while, afterwards, it exponentially decreases along the beam direction.”

The specific interactions of charged particles with matter can help in sparing the OARs, while keeping the same Planning Treatment Volume (PTV) coverage (7, 8) and has been exploited since twenty years in PT which uses protons and carbon ion beams (9, 10). However, an expensive and complex technology with dedicated facilities is needed to exploit proton and carbon beams, resulting in a high initial investment for a PT clinical centre. Furthermore, in PT treatments safety margins have to be introduced to account for uncertainties related to the patient positioning, organs movement, changes in the patient morphology with respect to the imaging used to plan the treatment. The eventual use of electrons, characterised by a broader dose deposition distribution with respect to protons and carbon ions, results in treatment plans that are more robust against range uncertainties, minimising the need of specific safety factors.

Very High Energy Electron (VHEE) beams have thus been explored in literature for the treatment of deep seated tumours. When comparing VHEE treatments with standard RT ones, the conformality of the former absorbed dose distribution to the PTV is comparable with the latter one only at the expense of using a large number of electron fields (order of tens, at least) and a beam energy larger than 100 MeV (11). Both requests contributed, so far, to make the VHEE solution more expensive and technologically challenging for a clinical centre with respect to IMRT or other photon-based RT solutions.

This landscape could change in the near future. Several pre-clinical studies recently claimed that the toxicity in healthy tissues related to tumour treatments can be significantly reduced, while keeping the same efficacy in cancer killing, if the dose rate is radically increased (~50 Gy/s, or even more) with respect to conventional treatments (~0.01 Gy/s). Such effect is known as the FLASH effect (12–14). Recently the first patient affected by a highly resistant skin lymphoma was treated using the FLASH irradiation approach and low energy electron beam (40 MeV) with a promising result (15).

The implementation of FLASH beams in clinical centres still has to overcome significant technical challenges. While the electron beams (in particular the low energy ones, used in Intra-Operative Radiation Therapy) have been already delivered with FLASH intensities, the implementation of FLASH RT with photons is still ongoing (13). Concerning protons, the challenge is related to the development of an ultra-fast energy changing technique capable of fully covering the PTV volume at the dose-rates needed to trigger the FLASH effect (16). To this aim approaches that make use of mono-energetic high energy proton beams have also been proposed.

**Figure 1:**
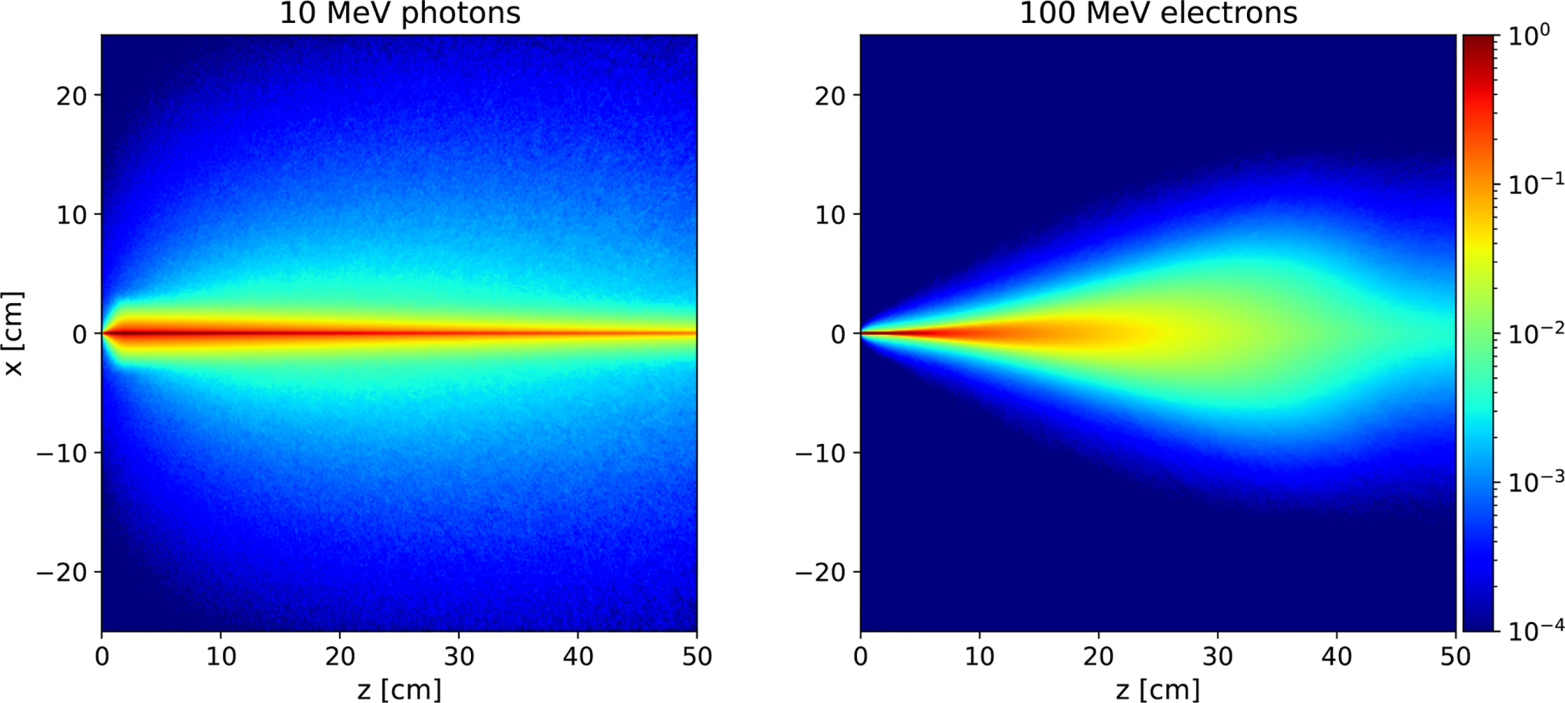
Absorbed dose distributions inside water generated from monochromatic beams of 10 MeV photons (Left) and 100 MeV electrons (Right).

**Figure 2:**
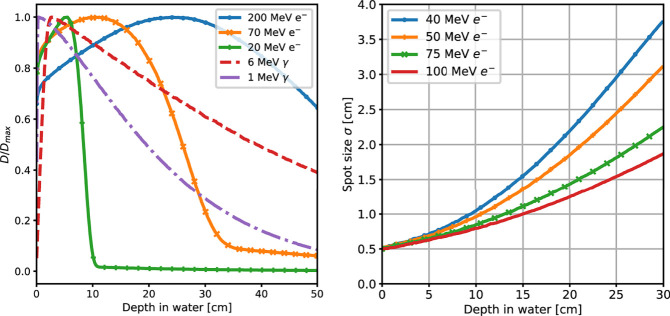
(Left) Absorbed dose depth distributions, normalised to their peak value, obtained from a MC simulation of mono-energetic electron and photon beams interacting with water. The simulated energies are in the range of interest for RT applications. (Right) MC simulation results for the lateral spread of VHEE beams of different energies as a function of their depth in water.

An example of the different interactions with matter of photons and electrons that can be used to treat deep seated tumours is reported in **Figure 1**, showing the energy deposition profile of 10 MeV photons and 100 MeV electrons. The electron dose distribution is characterised by a broad peak that is better suited for the treatment of deep tumours in comparison to photons (see **Figure 2**, left). An important feature of the electron dose distribution is its widening as the beam penetrates into the patient, in particular for lower energy, as shown in **Figure 2** (right). If the energy of the electron beam is increased well beyond 100 MeV, the beam broadening becomes negligible. As a consequence, using several fields a treatment comparable or better than the standard RT ones can be obtained (7, 8). In the past, such energies were not considered suitable for the clinical practice due to cost, complexity and space encumbrance. However, some of these issues can nowadays be addressed due to recent advances that allow to design compact accelerators matching the required energy with fields up to 50 MeV/m and with very high intensities (17–19).

In this manuscript we focused on the use of VHEE beams, with a low number of fields and taking into account the FLASH effect potential. Recently, a simulation has been carried out to study the feasibility of high energy (40 MeV) electrons FLASH irradiation of paediatric brain tumour with promising results (20). We decided, instead, to use real prostate IMRT treatments to benchmark the FLASH VHEE potential.

## 2 Methods

To evaluate the potential of VHEE treatments implementing the FLASH effect, we compared the VHEE results with three clinical cases (PZ1, PZ2, PZ3) of prostate tumour treated using IMRT where the irradiation made use of five to seven fields. The tumour (PTV) coverage and the dose absorbed by the OARs have been compared with the results obtained in real IMRT cases, from patients treated at the Department of Radiotherapy, Policlinico Umberto I, “Sapienza” University of Rome, by means of Dose Volume Histograms (DVH).

The clinical target volume consisted of prostates without seminal vesicles. Patients had intermediate-risk prostate cancer and were treated with conventionally fractionated EBRT. PZ1 was treated using 7 fields and had a medical prescription of 78 Gy, PZ2 and PZ3 were treated using 5 fields and had prescriptions of in 78 and 76 Gy respectively. The dose was delivered in 2 Gy fractions, resulting in a total of 39 fractions for PZ1 and PZ2 and 38 fractions for PZ3. A 6 MV-ONCOR Linear Accelerator, produced by Siemens, was used for the treatment.

In the VHEE treatment plan optimisation the calculation of the expected absorbed dose in the patient tissues has been performed using a MC simulation based on the patient Computed Tomography (CT). In the following we present in detail how the MC simulation was performed, how the FLASH effect was implemented and how the plan was optimised using the biological dose distributions.

### 2.1 Absorbed Dose Evaluation

In our study we used the FLUKA (21, 22) MC software to evaluate the absorbed dose in VHEE treatments. To compute the dose released by an electron of the beam FLUKA needs, as input, the CT information, the points from which the electron is originated with respect to the CT position, and the beam features (energy, spread) at the production point. The latter information depends on the characteristics of the accelerator and beam delivery technologies. Currently there are a lot of attempts aiming at developing compact high intensity electron beams of high energy. The advent of ‘C-band’ accelerating structures (11, 17–19) allows to reach the 50 MeV/m accelerating field suited for the VHEE implementation in a clinical centre. In our simulations the beam characteristics are those that are common to all the proposed conventional electron linac solutions with energy greater than 50 MeV (23, 24): the beam has transverse size (*0*~mm) and divergence (*0*~ mrad). We also restricted ourselves to consider only the relatively low energy range of VHEE: 70 to 130 MeV. We remark that the exact value of the spot size and of the angular divergence have a negligible effect on the dose distribution as the multiple scattering undergone by the electrons inside the patient tissues dominates.

We also assume an active scanning technique (14), similar to the one currently adopted in PT, where each field id made of equal energy pencil beams that are magnetically deflected to cover all the field region. The active scanning implementation is much easier for electron beams with respect to protons due to their significantly reduced magnetic rigidity. The possibility to let each PB energy to vary, as currently done in the active scanning system of PT, leaves margins of improvement in the treatment optimisation for future studies.

**Figure 3:**
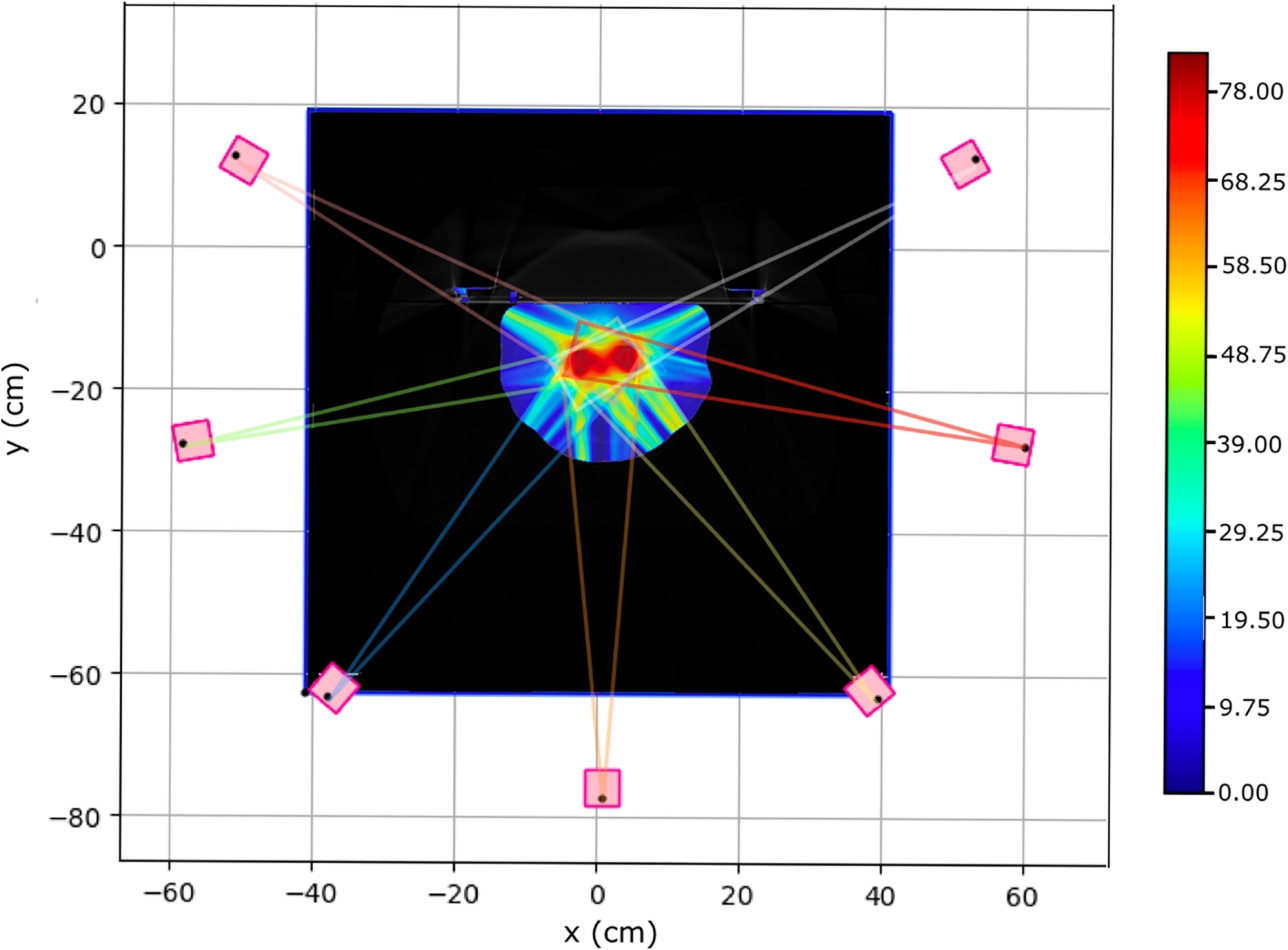
Pictorial view of the prostate target volume of PZ1 (highlighted using a black contour line) with superimposed the seven VHEE different fields. The lines in different colours are showing the maximum aperture for each field. Several PBs have been used to span and cover each field, in each slice: only the most external ones are shown.

As a simple starting point for our study of the VHEE treatment planning we considered the same IMRT fields and entry points. The photon IMRT approach is intermediate between the 2 opposite fields usually adopted in PT and the very large number of fields (16 or more) so far explored in VHEE applications (11). Each field was built using pencil beams with an initial radius of 5 mm (RMS) with directions determined to ensure a proper PTV coverage. **Figure 3** shows a slice of the PZ1 CT with the prostate highlighted using black contour lines. The optimised absorbed dose distribution when using VHEE is superimposed to the CT together with a sketch illustrating the maximum aperture for each of the seven fields, in the same plane of the CT slice.

The energy of each field used in the MC simulation, listed for the three patients in **Table 1**, has been determined aligning the dose peak to the PTV.

**Table 1:** Energies of the electrons belonging to a given field used to perform the treatment simulation for the three patients under study.

Energy [MeV]							
	Field 1	Field 2	Field 3	Field 4	Field 5	Field 6	Field 7
PZ1	70	130	130	120	120	130	130
PZ2	70	120	130	130	120	–	–
PZ3	110	130	100	130	100	–	–

Energies up to 130 MeV were considered, in order to place the electrons Bragg peak in the PTV region.

In addition to this approach in which the energy of each field can vary in the 70–130 MeV range, we have also performed a study of PZ1 in which the energy of all fields was set to 70 MeV: in that case, the potential of the FLASH effect in reducing the maximum energy needed to ensure the proper PTV coverage was tested against the other option in which the reduction of the dose lateral spread was achieved by means of an increased field energy.

### 2.2 Dose Modifying Factor

To quantify the reduced radiation-induced toxicity in normal tissues in the FLASH approach, when comparing to conventional RT, the DMF was introduced (25). The DMF values have been computed considering, as input, the significant number of papers documenting the evaluation of the healthy tissues ‘sparing’, in FLASH conditions, and looking at the reduced onset of after-treatment toxicities in different organs (26, 27). Concerning the PTV, we assumed that the FLASH irradiation maintains the same treatment efficacy as demonstrated in *in vivo* studies.

In this first explorative study, the absorbed dose in FLASH RT treatments has been computed using the same DMF values for all the OARs and the normal tissue. The treatment plans have been optimised using different DMF values: 0.6, 0.7, 0.8, 0.9 and 1. The last one has been used in order to provide VHEE results in conventional delivery mode. We chose the DMF values to explore sound and conservative sparing scenarios according to experimental findings: from the most conservative in which the DMF is set to 0.9, to the best case scenario in which a DMF of 0.6 could be reached.

### 2.3 Treatment Optimisation

Once the absorbed dose maps have been obtained by MC computation for each PB in the treatment plan, the fluence of each PB is optimised to ensure the required PTV coverage while sparing the OARs. The mathematical details are similar to the ones implemented in the active scanning TPS used in PT (28). The implemented algorithm defines a cost function made of two terms: the first constrains the biological dose inside the PTV to the goal value for each fraction (2 Gy, in our case) while the other is related to the OARs and it is activated whenever a threshold in the OAR voxels is surpassed. In the latter case, the optimiser uses the threshold as target dose inside the OAR for the over-threshold voxels. To take into account the huge volume difference (and hence number of voxels) between the PTV, the OARs and the normal tissues crossed by the beam, a voxel weighing in the cost function is implemented. Furthermore, to account for the different priorities when minimising the cost function, each PTV voxel has a weight equal to one while the OARs and normal tissue voxels enter the cost function multiplied by a weight equal to 10%. Such weighing strategy is the same as the one currently implemented in standard software tools used for TPS planning (e.g. Pinnacle). The output of the optimisation process is the absorbed dose map used to compute the DVHs and compare with the standard IMRT treatments optimised using the Pinnacle RTP software.

The optimised plan needs to satisfy the dosimetric endpoints (associated to clinical side effects) characteristics of the pathology and organ under treatment. Dosimetric endpoints are either expressed as the fraction of volume (V_XX_) that absorbs a given amount of dose (XX Gy), or as the value of the maximum absorbed dose (and the associated volume) (29) and are shown in **Table 2** for all the OARs considered in a prostate treatment.

**Table 2:** Set of requirements that have to be satisfied by the planned treatment (29).

Organ	dosimetric constraints
Target volume	V_95%_ > 95_%_, never above 107%
Rectum	V_50_ <50%, V_60_ <35%, V_65_ <25%, V_70_ <20%, V_75_ <15%
Anus	V_30_ <50%
Bulbourethral Glands	$\bar{\text{D}}$ <50 Gy
Femurs	$\bar{\text{D}}$ <52 Gy, V_60_ <5%
Bladder	$\bar{\text{D}}$ <65 Gy, V_65_ <50%, V_70_ <35%, V_75_ <25%, V_80_ <15%

The checks are performed using the DVH information obtained using the Pinnacle software to evaluate the expected absorbed dose in the different patient tissues. $\bar{\text{D}}$ is the mean dose absorbed by a given organ or region. V*_XX_* is the fraction of volume of a given OAR (or PTV) that absorb a given (*XX* Gy) amount of dose. The requirement V*_XX_* <YY% should be read as: YY% of the referred organ or region must absorb less than *XX* Gy.

## 3 Results

**Figure 4:**
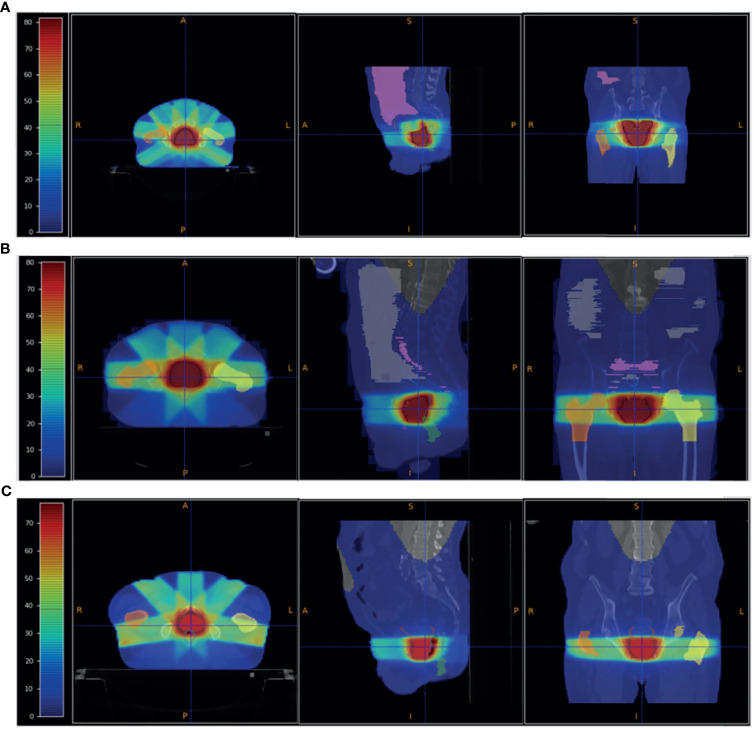
Patient PZ1 **(A)**, PZ2 **(B)** and PZ3 **(C)** CT overlapped with the dose map optimised using the Pinnacle TPS software for an IMRT treatment using respectively 7 (PZ1) and 5 (PZ2 and PZ3) photon beams. The OARs are shown: the femurs in yellow and orange, the bladder surface in brown, the rectum surface in dark blue. The PTV is shown in red. The absorbed dose related to the full treatment (39 fractions for PZ1 and PZ2, and 38 fractions for PZ3 of 2 Gy each) is shown.

VHEE treatments have been benchmarked against the results obtained using standard IMRT technique. **Figure 4** shows the PZ1, PZ2 and PZ3 IMRT treatment plans optimised using the Pinnacle software (RTP System Version 16, https://pinnacle-software.com/). Three different views of the absorbed dose distribution, overlaid on each patient CT, are shown.

**Figure 5:**
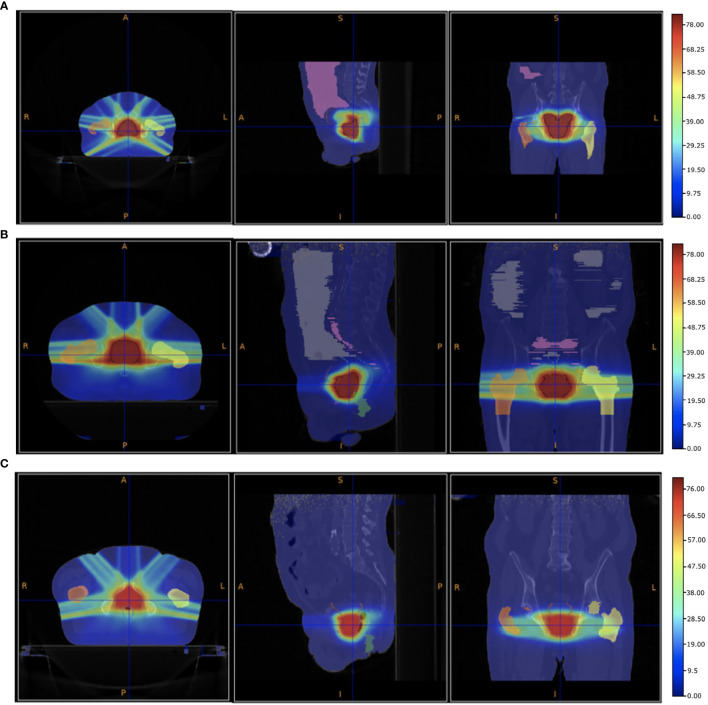
Patient PZ1 **(A)**, PZ2 **(B)** and PZ3 **(C)** CTs overlapped with the biological dose maps optimised using the output of a FLUKA simulation using VHEE with energies listed in **Table 1** and a DMF of 1 (no FLASH effect). The OARs are shown: the femurs in yellow and orange, the bladder surface in brown, the rectum surface in dark blue. The PTV is shown in red.

In the VHEE treatment plan the biological dose maps, obtained multiplying the physical dose map by the DMF, have been used to compute each DVH and check the PTV coverage. The results are summarised in **Table 3**. The optimised dose maps obtained for an energy of 70 MeV and a DMF of 1 are shown in **Figure 5**. The same views and centering points of **Figure 4** have been chosen in order to ease the comparison with the results obtained with the conventional IMRT approach.

**Table 3:** Values of V*_xx_* and Deqs for the PTV and different organs obtained from a FLUKA MC simulation performed with electrons of different energies (see **Table 1** and different DMFs.

PZ1	DMF:	1	0.9	0.8	0.7	0.6
PTV	*V*_95%_	96.35%	98.3%	99.3%	99.86%	99.99%
	*V*_105%_	0.17%	0.03%	0.04%	0.05%	1.02%
Rectum	*V*_75_	0.86%	2.55%	4.1%	6.9%	7.85%
	*V*_50_	29.9%	24.3%	18.4%	12.3%	8%
Anus	*V*_30_	35.4%	33.7%	33.1%	33.8%	40.4%
Bulb	*D*_50_	42 Gy	40.5 Gy	38.8 Gy	37.3 Gy	36.1 Gy
Femurs	*D*_50_	16.2 Gy	14.8 Gy	14.1 Gy	14.1 Gy	13.7 Gy
Bladder	*D*_50_	38.2Gy	36.7 Gy	35.6 Gy	33.8Gy	32.4 Gy
	*V*_70_	17%	10.6%	9.4%	9.4%	9.4%
	*V*_65_	20%	17.3%	9.4%	9.4%	9.4%
**PZ2**	**DMF:**	**1**	**0.9**	**0.8**	**0.7**	**0.6**
PTV	*V*_95%_	95.7%	97.3%	98.7%	99.7%	100%
	*V*_105%_	0.29%	0.08%	0.01%	0%	0%
Rectum	*V*_75_	0.8%	1.5%	2.3%	2.8%	3.3%
	*V*_50_	20%	17.1%	13.1%	6.8%	3.4%
Anus	*V*_30_	22.1%	20.9%	20%	19.4%	20.7%
Bulb	*D*_50_	12.3 Gy	14.9 Gy	22.5 Gy	22.6 Gy	19 Gy
Femurs	*D*_50_	26.8 Gy	26.1 Gy	25.3 Gy	22.5 Gy	18.3 Gy
Bladder	*D*_50_	45Gy	44.9 Gy	47.3 Gy	48.8Gy	45.8Gy
	*V*_70_	19.6%	12.2%	12%	12%	12%
	*V*_65_	25.2%	19.9%	12%	12%	12%
**PZ3**	**DMF:**	**1**	**0.9**	**0.8**	**0.7**	**0.6**
PTV	*V*_95%_	96.1%	98%	99.2%	99.8%	100%
	*V*_105%_	0.02%	0%	0%	0.13%	0%
Rectum	*V*_75_	0.6%	1.3%	2.4%	5%	8%
	*V*_50_	34.7%	30.5%	25.6%	16.6%	9.7%
Anus	*V*_30_	0%	0%	0%	0%	0%
Bulb	*D*_50_	38.9 Gy	38.2 Gy	36.8 Gy	36.2 Gy	36.5 Gy
Femurs	*D*_50_	10.2 Gy	11.4 Gy	11.3 Gy	10.5 Gy	9.4 Gy
Bladder	*D*_50_	22.3Gy	23.1 Gy	24.4 Gy	24.2Gy	22.7Gy
	*V*_70_	3.5%	1%	1.1%	1.1%	1.1%
	*V*_65_	7.3%	3.7%	1.1%	1.1%	1.1%

Results for all the three patients and DMF values explored are reported. All the obtained values satisfy the requirements shown in **Table 2**, even for DMF 1. The bold values refer to the DMF values.

**Figure 6:**
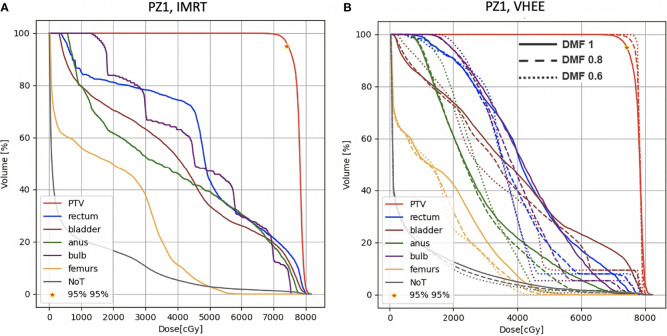
DVH histograms for the PTV and the OARs of PZ1. The biological dose relative to the normal tissue (NoT) is shown as well. **(A)** Results obtained with photons (standard IMRT, 7 fields) for the 39 fractions foreseen in the patient treatment (78 Gy in total). **(B)** Results obtained with electrons of different energies (see **Table 1**) and using different DMF values: the solid line shows results obtained without any FLASH effect, while dashed and dotted lines show the impact of a DMF equal to 0.8 and 0.6 respectively.

**Figure 7:**
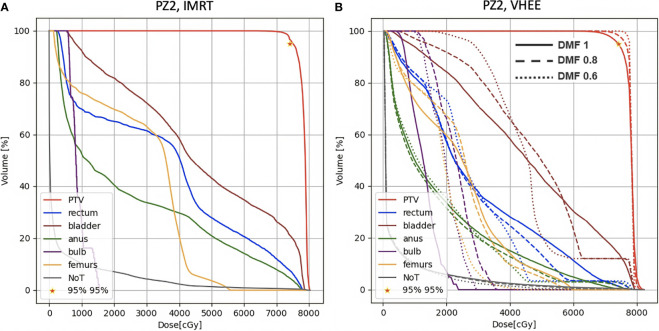
DVH histograms for the PTV and the OARs of PZ2. The biological dose relative to the normal tissue (NoT) is shown as well. **(A)** Results obtained with photons (standard IMRT, 5 fields) for the 39 fractions foreseen in the patient treatment (78 Gy in total). **(B)** Results obtained with electrons of different energies (see **Table 1**) and using different DMF values: the solid line shows results obtained without any FLASH effect, while dashed and dotted lines show the impact of a DMF equal to 0.8 and 0.6 respectively.

**Figure 8:**
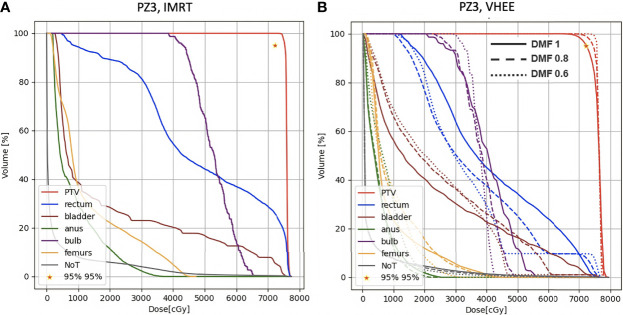
DVH histograms for the PTV and the OARs of PZ3. The biological dose relative to the normal tissue (NoT) is shown as well. **(A)** Results obtained with photons (standard IMRT, 5 fields) for the 38 fractions foreseen in the patient treatment (76 Gy in total). **(B)** Results obtained with electrons of different energies (see **Table 1**) and using different DMF values: the solid line shows results obtained without any FLASH effect, while dashed and dotted lines show the impact of a DMF equal to 0.8 and 0.6 respectively.

A more quantitative comparison can be performed studying the DVHs. **Figures 6**–**8** show the DVH comparison relative to the dose maps shown respectively in **Figures 4**, **5** for PZ1, PZ2 and PZ3.

In addition to the irradiation performed using fields with energies up to 130 MeV, that seems to provide results outperforming the IMRT even with DMF equal to one (no FLASH effect), it is also interesting to note that VHEE of 70 MeV with a DMF different from 1 are also able to provide a treatment that matches the requirements of **Table 1**.

**Figure 9:**
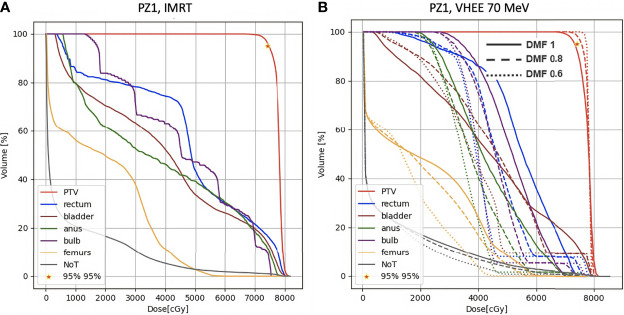
DVH histograms for the PTV and the OARs of PZ1. The biological dose relative to the normal tissue (NoT) is shown as well. **(A)** Results obtained with photons (standard IMRT, 7 fields) for the 39 fractions foreseen in the patient treatment (78 Gy in total). **(B)** Results obtained with electrons of 70 MeV and using different DMF values: the solid line shows results obtained without any FLASH effect, while dashed and dotted lines show the impact of a DMF equal to 0.8 and 0.6 respectively.

**Figure 9** shows the DVHs of PZ1 treated using VHEE of 70 MeV. In case of DMF equal to 1 it was not possible to satisfy the PTV coverage requirement. However, if a DMF of 0.8 is taken into account, as shown in **Figure 9** left, then the PTV coverage is ensured with an OARs sparing better than the one achievable with IMRT.

### 3.1 Prompt Positron Signal

The MC simulations allowed to also study the production of positrons. High energy electron beams produce prompt photons, and hence prompt positrons, that mainly annihilate at rest, producing two back-to-back photons of 511 keV energy, that can be exploited as Positron Emission Tomography (PET) signal. The production point of these ‘prompt’ PET photons has been studied to check the correlation of their spatial emission distribution with the absorbed dose in the treated volume.

**Figure 10:**
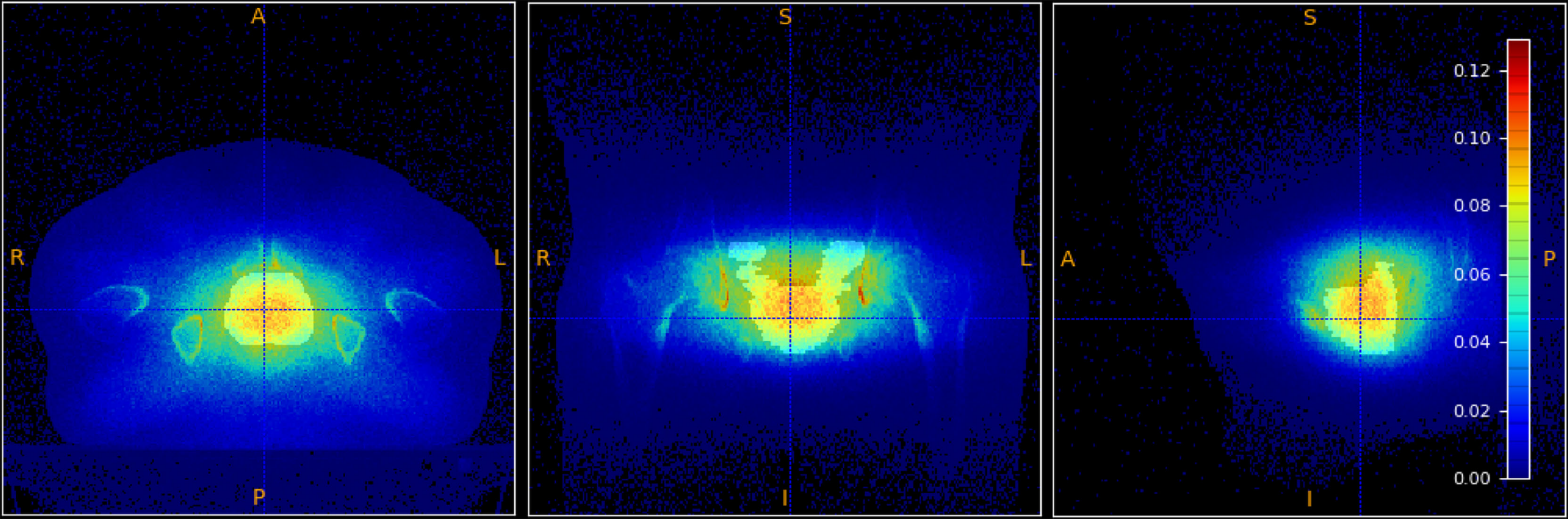
Absorbed dose in the PTV (shown in grey-scale) overlaid to the spatial emission distribution of prompt positrons.

From an experimental point of view, the PET signal detection in FLASH RT poses a significant technical challenge, in particular in terms of data acquisition rate capability. However the presence and significance of the PET signal could drive the needed R&D efforts to exploit it for treatment control or monitoring applications. **Figure 10** shows the correlation between the distribution of the absorbed dose in the PTV (in grey-scale) with, overlaid, the PET photons production. In the present work we report the existence of such correlation between the two distributions, leaving the quantitative modelling of such correlation to future papers.

## 4 Discussion

The obtained results demonstrate that it is possible, with the same number of fields used in IMRT and energies not exceeding 130 MeV, to optimise a treatment satisfying the PTV coverage constraints while achieving a better sparing of the OARs with respect to photon IMRT, even in absence of FLASH effect.

Summarising, the comparison of the results obtained with VHEE (**Figure 5** and **Table 3**) and with conventional RT (**Figure 4**), and looking at the DVHs shown in **Figures 6**–**8**, suggest that:

- electrons in the energy range considered in this manuscript (from 70 to 130 MeV) can be used to treat a prostate cancer using the IMRT irradiation scheme (same number of fields) at conventional dose rates. In this case a PTV coverage similar to the IMRT is obtained by adapting the mean energy of each field to the value needed to centre the Bragg peak in the PTV; the sparing of the OARs slightly outperforms the IMRT;
- when a DMF different from 1 is implemented, the organs sparing and the PTV coverage improve significantly. This will definitely help in reducing the electrons energy needed to achieve the same treatment efficacy: with a DMF of 0.8 a prostate cancer treatment with electrons of 70 MeV would be competitive with IMRT;
- such promising results are obtained without any field entrance point optimisation that could exploit the differences in the lateral dose distributions of photons and electrons to additionally increase the gain with respect to IMRT;
- the results obtained for rectum and bladder are particularly interesting especially in view of hypo-fractionated treatments that would probably happen in FLASH therapy applications. VHEE are significantly reducing the acute dose to these organs and are hence better suited for applications in which the dose per fraction increases.

These results have been achieved using solid and minimal assumptions: the IMRT irradiation geometry was not changed and it was assumed that the PTV could be covered using the same active scanning methods already implemented in PT treatments (14). We also considered, conservatively, a treatment where all the pencil beams of a given field have the same energy. These assumptions lead unavoidably to a sub-optimal VHEE performance but left open the possibility for further improvements. This consideration is particularly important for the DMF = 1 result, where no FLASH effect has been assumed. Other beam delivery techniques, for example the case in which a wide mono-energetic beam is used to cover the whole are under treatment using a collimator to properly shape the beam so to achieve the required dose conformity to the PTV, will be explored in future papers. That approach simplifies the fulfilment of the dose rate requirement over the whole PTV but poses significant technical challenges, since the FLASH intensity has to be reached on a large volume simultaneously, and implies the use of a passive media that produce additional unwanted secondary radiation whose impact needs to be carefully taken into account.

Concerning the FLASH effect impact, there are few important points that have to be stressed. There is a significant experimental effort currently aiming at determining what is the minimum absorbed dose needed to trigger the effect and what is the maximum time allowed for the irradiation of a given voxel or volume to ensure that an additional sparing is obtained with respect to conventional RT. The results of these studies will definitely affect the beam delivery and the fractionation schemes that will adopted in the future of FLASH RT. In this context, it is clear that the largest benefits will come from those pathologies in which large (≥ 5–6 Gy) doses per fraction are allowed.

In particular, some of the simplifications that we have used when modelling the FLASH effect will have to be abandoned in the future. In this contribution, a constant DMF has been applied to all the healthy tissues and OARs. However, it is unlikely that hypo-fraction scheme will ensure that all the OARs will receive a dose large enough to trigger the FLASH effect. In that sense, for the OARs receiving small doses, the impact of the FLASH effect might be overestimated. At the same time it is also true that the OARs closest to the PTVs, that are mostly affected by the dosimetric constraints, would have the largest benefits from the FLASH effect. A DMF modelling capable of accounting for the dependence on each fraction absorbed dose will be needed to refine the performance evaluation obtained in this work. However we can still conclude that the crude approximation made in our study already hints that impressive results can be obtained even in case of a modest (10–20%) gain in the healthy tissues sparing.

## 5 Conclusions

The results presented in this manuscript and obtained on real cases of prostate cancer demonstrate the potential for treating deep seated tumours with external VHEE beams provided by a delivery system implementing the active scanning technique. The implementation of VHEE RT could allow the proper PTV coverage, while achieving a better OARs sparing, with the additional benefit of reducing the impact of range uncertainties (large in PT) in the treatment planning.

Consistently with what already obtained elsewhere (8), without the FLASH effect the energy needed to deliver treatments of comparable efficacy with respect to IMRT or VMAT must be greater than 100 MeV. However, if the FLASH effect is taken into account, lower energies can be exploited opening a new landscape for the clinical implementation of VHEE treatments.

A better evaluation of the potential benefits of FLASH irradiation will be performed once experimental data will be available to determine the absorbed dose threshold needed trigger the effect and the impact of beam delivery strategies. Preliminary results are however promising showing a clear potential for VHEE deep tumor irradiation when delivered at ultra-high dose rates.

## Data Availability Statement

The raw data supporting the conclusions of this article will be made available by the authors, without undue reservation.

## Author Contributions

ASar, PM, MF, GF, MP, ASch, and VP wrote the main manuscript text. AS, MS, GF, MM, LR, DR, and ASch prepared the figures. ASar, PM, FM, CF, and VT provided the information about the dosimetric constraints that plans have to satisfy and the treatment plan with photons. GB, PM, YD, IM, RM, and SM defined the dose modifying factor modeling inside the optimisation algorithms. LP, ASch, ASci, and ASar provided the information about the beam model to be used in the MonteCarlo simulations. MF, VP, LR, DR, ASar, ASch, GT, MT, and AT implemented the simulation, optimization algorithms, and performed the data analysis. All authors contributed to the article and approved the submitted version.

## Conflict of Interest

The authors declare that the research was conducted in the absence of any commercial or financial relationships that could be construed as a potential conflict of interest.

## Publisher’s Note

All claims expressed in this article are solely those of the authors and do not necessarily represent those of their affiliated organizations, or those of the publisher, the editors and the reviewers. Any product that may be evaluated in this article, or claim that may be made by its manufacturer, is not guaranteed or endorsed by the publisher.
